# The Use of Both Therapeutic and Prophylactic Vaccines in the Therapy of Papillomavirus Disease

**DOI:** 10.3389/fimmu.2020.00188

**Published:** 2020-02-18

**Authors:** Anna Rosa Garbuglia, Daniele Lapa, Catia Sias, Maria Rosaria Capobianchi, Paola Del Porto

**Affiliations:** ^1^Laboratory of Virology, “Lazzaro Spallanzani” National Institute for Infectious Diseases, IRCCS, Rome, Italy; ^2^Department of Biology and Biotechnology “C. Darwin,” Sapienza University, Rome, Italy

**Keywords:** human papillomavirus, immune response, cancer, prophylactic vaccine, therapeutic vaccine

## Abstract

Human papillomavirus (HPV) is the most common sexually transmitted virus. The high-risk HPV types (i.e., HPV16, 18, 31, 33, 35, 39, 45, 51, 52, 56, 58, 59) are considered to be the main etiological agents of genital tract cancers, such as cervical, vulvar, vaginal, penile, and anal cancers, and of a subset of head and neck cancers. Three prophylactic HPV vaccines are available that are bivalent (vs. HPV16, 18), tetravalent (vs. HPV6, 11, 16, 18), and non-avalent (vs. HPV6, 11, 16, 18, 31, 33,45, 52, 58). All of these vaccines are based on recombinant DNA technology, and they are prepared from the purified L1 protein that self-assembles to form the HPV type-specific empty shells (i.e., virus-like particles). These vaccines are highly immunogenic and induce specific antibodies. Therapeutic vaccines differ from prophylactic vaccines, as they are designed to generate cell-mediated immunity against transformed cells, rather than neutralizing antibodies. Among the HPV proteins, the E6 and E7 oncoproteins are considered almost ideal as targets for immunotherapy of cervical cancer, as they are essential for the onset and evolution of malignancy and are constitutively expressed in both premalignant and invasive lesions. Several strategies have been investigated for HPV therapeutic vaccines designed to enhance CD4^+^ and CD8^+^ T-cell responses, including genetic vaccines (i.e., DNA/ RNA/virus/ bacterial), and protein-based, peptide-based or dendritic-cell-based vaccines. However, no vaccine has yet been licensed for therapeutic use. Several studies have suggested that administration of prophylactic vaccines immediately after surgical treatment of CIN2 cervical lesions can be considered as an adjuvant to prevent reactivation or reinfection, and other studies have described the relevance of prophylactic vaccines in the management of genital warts. This review summarizes the leading features of therapeutic vaccines, which mainly target the early oncoproteins E6 and E7, and prophylactic vaccines, which are based on the L1 capsid protein. Through an analysis of the specific immunogenic properties of these two types of vaccines, we discuss why and how prophylactic vaccines can be effective in the treatment of HPV-related lesions and relapse.

## Introduction

Despite the introduction of prophylactic vaccines, the incidence of human papillomavirus (HPV)-related tumors remains high (1), particularly in developing countries of the sub-Saharan region. The current HPV prophylactic vaccines have indications for use in women up to the age of 45 years, but they are predominantly administered to adolescent of 9–15 years. As most cancers develop decades after an initial HPV infection, the impact of this vaccination program will be seen in the long-term. Therefore, setting-up a therapeutic vaccine that can provide results similar to surgical treatment or chemotherapy represents a challenge for the eradication of HPV-induced tumors. However, therapeutic vaccines are not yet available for clinical practice.

Several studies have suggested that administration of HPV prophylactic vaccines after surgical treatment of high-grade cervical intraepithelial neoplasia (CIN2-3) can be considered as an adjuvant to prevent HPV reactivation or reinfection. The relevance of prophylactic vaccines has also been demonstrated in the management of genital warts, although clinical studies have not delivered univocal results to date.

In this review, the HPV-related tumors and the life cycle of HPV are described, to better understand the characteristics of the different viral proteins that are targeted by prophylactic and therapeutic vaccines. Then, we summarize the leading features of prophylactic and therapeutic vaccines that target the L1 and E6, E7 oncoproteins, respectively. Finally, through an analysis of the specific immunogenic properties of these two types of vaccines, we discuss how prophylactic vaccines can be effective for the treatment of HPV-related lesions, and for prevention of HPV-related relapse.

## HPV Prevalence

Human papillomavirus is the main agent of sexually transmitted diseases, and it can cause cancer in different anatomical districts with different prevalences (2). The highest proportion of HPV attribution as responsible for a cancer is for the cervix, where >99% of specimens are HPV-positive. In 2012, HPV-invasive cervical cancers reached >500,000 cases, which resulted in ~250,000 deaths around the world^1^. HPV-related cancers are differently distributed across genders: among women, 8.6% of cancers are linked to HPV infections, while in men, <1% of cancers are attributable to HPV^2^^,^^3^. Differences are also observed in the geographic distributions of invasive cervical cancers: more than 85% occur in developing countries, where cervical screening programs and HPV vaccination campaigns are rarely available^4^ (3, 4). HPV infection in a cervical site is frequently asymptomatic, and >90% of these resolve within 2 years without medical intervention (5), apparently through rapid immune clearance. However, the protective power of natural anti-HPV antibodies and the duration of immunity after infection are not fully understood. Also, only 50–60% of women show detectable anti-HPV antibodies after infection (6).

There are 15 high-risk (HR)-HPV genotypes that can lead to cancers of the cervix, anus, penis, vagina, vulva, and oropharynx (i.e., HPV16, 18, 3, 33, 35, 39, 45, 51, 52, 56, 58, 68, 73, 82) (7). The relevance of HPV to each of these individual cancers is now considered.

### Cervical Cancer

Overall, 90% of cervical cancers are attributed to HR-HPV types. HPV16 and HPV18 are the most prevalent in invasive cervical cancer, where they account for 62.5 and 15.7% of cases, respectively (8). HPV-associated cancers include cervical squamous cell carcinoma (70%), cervical adenocarcinoma (25%), and mixed histology tumors (7)^5^. An immunocompromised status represents a risk factor for cervical dysplasia, as well as for latent reactivation of HPV at genital sites. Patients with human immunodeficiency virus (HIV) infection have a 5-fold greater risk of acquiring HPV-associated cervical cancer than those without HIV infection. Precancerous (squamous) intraepithelial lesions are categorized as low-grade (LSIL) and high-grade (HSIL) (9).

### Anal Cancer

Anal intraepithelial neoplasia (AIN1-3) represents the precursor of invasive anal cancers, where 65% are cervical squamous cell carcinomas. For both sexes, 88–94% of these cancerous lesions are positive for HPV DNA, with HPV16 as the most commonly detected (~87% of HPV-positive tumors), while only 9% of these anal cancers harbors HR-HPV18^6^.

Annually, about 18,000 women are diagnosed with anal cancer worldwide, and this cancer is more frequent in women than in men (10). Furthermore, anal cancer incidence is increasing, which appears to be due to changes in sexual risk factors for HPV transmission (11). Persistent anal HPV infection is the major cause of anal cancer^7^ (12). Women with a history of cervical cancer and cervical intraepithelial neoplasia grade 3 (HSIL) are also at increased risk of anal cancer. Cervical HR-HPV–positivity is associated with anal HR-HPV prevalence. In a study carried out by Lin, anal HR-HPV prevalence was significantly higher in cervical HR-HPV–positive women (43%) vs. cervical HR-HPV–negative women (9%) (13). These associations were even stronger for HPV16-positivity: in cervical HPV16-positive women, anal HPV16 prevalence was 41%, while in the HPV16-negative cervical group, anal HPV16 prevalence was 2%.

In men, the risk of anal cancer development is strictly related to sexual behavior and HIV immune status (14).

### Penile Cancer

Approximately 50% of penile cancers can be attributed to HPV infections, although HPV also infects healthy subjects without progressing to neoplasia. In a British study carried out among 43 couples, 69% of the men were HPV-positive in the uro-genital tract (15). Another study in the USA showed similar high levels of HPV prevalence, with 65.5% of the men HPV-positive: 51.2% of them harbored at least one typed HPV, and 14.3% of them were positive for an unclassified HPV infection (16). HPV clearance is observed in 75% of cases within 1 year (17). HPV-positivity is greater in penile intraepithelial neoplasia (PIN 1,2,3), which is considered the precursor of penile cancer, and in basaloid histological neoplasia (range, 75–80%) than in invasive cervical squamous cell carcinoma (range, 30–60%). HPV16 and HPV18 are the HPV types that are most frequently associated with all types of penile cancers^2^.

### Vulvar Cancer

It is estimated that 40–50% of vulvar cancers are associated with HPV. Overall, vulvar cancers represented 3% of gynecologic cancers in 2002 (18), and 60% of were observed in developed countries (18, 19)^8^. Vulvar intraepithelial neoplasia (VIN) is considered a precursor of vulvar squamous cell carcinoma, which represents >90% of vulvar cancers (20). The World Health Organization recognizes a three-grade system of VIN (i.e., VIN1-3), and VIN3 is considered as a precursor of invasive vulvar cancer. However, until recently, VIN2-3 had been considered as HSIL, and VIN1 (or LSIL) is no longer used, as there is misclassification of these low-grade lesions: these are often actually condilomata acuminata with transient HPV infection, or an inflammatory status of the vulva (21). VIN can be caused by two distinct etiological agents: HPV, which is linked to the usual form of VIN (uVIN), and differentiated VIN (dVIN) is associated with lichen sclerosus (22, 23). Generally, uVIN is common among young women, while dVIN is frequently seen for post-menopausal women. In VIN3/HSIL lesions, HPV16 had been detected in >91% of cases (24).

### Vaginal Cancer

Cancer of the vagina is rare, and it accounts for only 2% of gynecological neoplasia. Nevertheless, from 2000 to 2015 there was an increase in vaginal cancer, which corresponded to 0.4% of vulvar carcinomas in the USA^9^. Vaginal intraepithelial neoplasia (VAIN) is considered a pre-malignant lesion. Previously, a three-tiered classified was used (VAIN1-3) according to epithelial involvement. In 2014, the World Health Organization revised this classification by substituting VAIN2-3 with HSIL, and VAIN1 with LSIL (25). About 82% of high-grade lesions (i.e., vaginal intraepithelial lesions, VAIN3, HSIL) and 91% of invasive vaginal carcinomas test positive for HPV DNA and/or HPV antibodies. HPV16 is the most prevalent HPV type in HSIL and vaginal cancers^2^ (26–29).

### Head and Neck Cancer

The most common head and neck cancers are squamous cell carcinomas (HNC) and they include neoplasia of the oral cavity, tongue, tonsils, oropharynx, hypopharynx, and larynx. The HR-HPV types most frequently detected in head and neck cancers are HPV16, followed by HPV18^2^ (30, 31). Head and neck cancers are frequent in southern-central Asia (32); however, an increase in head and neck cancer incidence has been seen over recent years in developed countries (33) and among Caucasian men (34). Tonsillectomies can increase the overall survival rates of patients with diagnosis of tonsil carcinoma (35), but it does not influence the overall risk of oropharyngeal cancer (36).

A reduction in HPV-positive oropharyngeal cancer is observed in people with a specific genetic locus in the human leukocyte antigen region (HLA-DRB1^*^1301-HLA-DQA1^*^0103-HLA-DQB1^*^0603) (37). This protective effect might involve increased immune targeting of HPV-infected cells through the major histocompatibility complex haplotype binding to HPV peptides, resulting in a strong CD4^+^ T-cell response (38).

### Respiratory Papillomatosis

Different annual incidences of respiratory papillomatosis have been reported in different countries. For example, in Denmark, similar incidence has been reported in children (juvenile onset) (3.6/100,000 children) and in adults (3.9/100,000 adults) (38), while in the USA the annual respiratory papillomatosis incidence is 3-fold higher in children than in adults (4.6 vs. 3.9/100,000 children/adults) (39).

HPV6 and HPV11 are the main genotypes detected in respiratory papillomatosis. As spontaneous regression is rarely observed, surgical treatment is necessary to prevent progression of the lesions. Moreover, recurrence of papillomatosis is often observed, and retreatment is needed in most cases, which comes at a high economic burden (40). Although no structured trials have been carried out to date, HPV vaccine administration prior to the onset of sexual behavior might have a positive impact on prevention of respiratory papillomatosis in adulthood.

### Genital Warts

Human papillomavirus infection can not only cause cancer, but also benign genital warts. These are very diffuse in the young and in adults, with prevalence from 4 to 11% (41–43). Treatment of genital warts includes therapies with imiquimod and podophyllotoxin, or surgical procedures, or cryotherapy and tricloroacetic acid. These medical interventions represent high costs for both private insurance (44) and health systems (45).

Condylomas were classically considered a benign lesion, with the exception of Buscke-Lowenstein tumors. This large tumor can undergo local invasion and can transform into anal cervical squamous cell carcinoma (46).

## Viral Characteristics and Immune Responses

### Life Cycle of HPV

Human papillomaviruses are non-enveloped icosahedral viruses that consist of 72 capsomers and are 55 nm in diameter. The viral genome is a circular double stranded DNA of ~8,000 bp in length.

**Figure 1:**
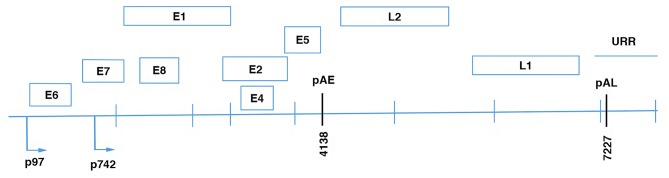
Linear diagram of HPV16 genome showing the eight open reading frames (ORFs), the early (pAE), and the late (pAL) polyadenilation sites; p97, promoter of E6 and E7 viral oncoproteins; p670, promoter of the late proteins.

According to the time-regulated expression of proteins during the viral cycle, three functional genome regions can be distinguished: (i) the early region that encodes the E1, E2, E4, E5, E6, E7, E8 viral proteins that have regulatory functions in infected epithelial cells; (ii) the late region that encodes the two viral capsid proteins L1 and L2; and (iii) the long control regions (also known as upstream regulatory regions) that contain *cis*-acting regulatory sequences that are involved in the control of viral replication and post-transcriptional phases (Figure 1).

Different viral proteins are expressed in different layers of the epithelium. The early proteins are expressed in basal epithelial cells, while the late proteins are expressed in the granular layer that includes more differentiated cells, and from where the virus is assembled and released. E1 is an ATPase helicase that is involved with E2 in viral replication and transcription regulation. The E1 and E2 complex interacts with the long control region ori site, which is considered to be the origin of HPV DNA replication (47–53). In the initial phase of infection, the HPV DNA genome is in the episomal form. It shows low amplification activity and there are ~100 copies/cell (54). E2 ensures episomal maintenance of the HPV genome through interactions with other cellular factors. For example, Bromo-domain protein 4 (BRD4) is a mitotic chromosome-associated protein that is a critical binding partner for E2 for this activity (55). BRD4 and E2 co-localize on condensed mitotic chromosomes, and mediate episome segregation (55). E2 also regulates transcription of the E6 and E7 oncoproteins, the expression of which depends on an early promoter. E1 to E4 are encoded by a spliced RNA, and along with E5, they are translated under early promoter control in undifferentiated cells, and they appear to facilitate efficient productive replication in differentiating cells (56, 57).

E6 and E7 are small proteins of about 150 and 100 amino acids, respectively. The E6 oncoprotein acts through its PDZ-binding motif, which promotes its interactions with PDZ domains in multidomain proteins, to alter their functionality. These PDZ domains are present in many multidomain proteins that regulate key steps in the cellular processes of apoptosis, adhesion, and polarity (37–43, 58). E6 also impairs the activity of the p53 protein, which prevents DNA damage accumulation through induction of DNA repair, cell-cycle arrest, or apoptosis, which leads to transformation of HPV chronically infected cells.

The main targets of E7 are the pRB, p107, and p130 proteins, which are components of a complex that can repress the E2F transcription factor (59, 60). When E7 interacts with pRB, p107, and p130, it induces their degradation, and so E2F is free to activate genes such as cyclins A and E, to promote transition from G1 to S-phase of the cell cycle (61). The productive viral cycle also includes the synthesis of the late proteins L1 and L2 in the suprabasal epithelial cell layers, and this step is characterized by a change in mRNA splicing (62, 63). Icosahedral virions are composed of 360 L1 proteins that are organized in pentamers, each of which is associated with one monomer of L2. The productive life cycle is completed when the virions self-assemble, after packaging of the amplified HPV DNA genome, with the viral particles then shed from the epithelial cell layers (64).

### Natural Immune Response

The immune response has an important role in clearing most HPV infections, although sometime the virus cannot be eliminated and can persist for several years, which represents a risk factor for neoplasia development (65). HPV-associated neoplastic progression is linked to dysregulated expression of the early viral genes. Specifically, increased expression of the E6 and E7 proteins in the basal epithelium leads to increased cell-cycle entry and loss of differentiation across the epithelium. The main cause of dysregulated HPV gene expression is integration of the viral genome into the host chromosomes (66). HPV DNA integrates randomly into the host DNA. During this process, the viral DNA can often be broken at any position within the E1-E2 region, with the loss of E2 function, and the consequent overexpression of E6 and E7 that promotes cellular transformation (67–69). However, a proportion of cervical cancers are associated with episomal DNA only. In such cases, the E2 open reading frame integrity is maintained, and this protein is expressed throughout the progression of the malignancy.

In natural infections, both humoral- and cell-mediated immune responses are induced. Genital infection with oncogenic HPV is common, but only a minority of infected patients develop epithelial lesions or cancer (70). Spontaneous clearance of an established infection is likely to be mediated by the cellular immune responses. Indeed, strong Th1 CD4^+^ T-cell responses that are specific for HPV16 E6, E7, and E2 have been frequently detected in peripheral blood mononuclear cells of healthy individuals (71). In contrast, responses against HPV16 E6, E7, and E2 have rarely been detected in patients with HPV16-positive genital lesions or antigen-specific proliferative responses that show a non-inflammatory cytokine profile (72, 73).

Similarly, effective HPV18-specific T-cell responses are only seen in healthy controls, and not in HPV18-positive patients (74). For the role of CD8^+^ T-cells in disease regression, a comparison of CD8^+^ T-cell responses to E6 and E7 using enzyme-linked immunospot assays in individuals with incident or prevalent HPV 16 or 18 infections did not show any significant difference in the frequency of positivity between these two patient groups (33 vs. 40%) (75). At variance with this, in CIN2/3 lesions, more CD8^+^ T-cells were seen for the epidermis of tissues that went on to regress (76). Also, large numbers of intraepithelial CD8^+^ tumor-infiltrating lymphocytes have been associated with an absence of lymph-node metastases in patients with large early stage cervical cancer (76). Taken together, these findings indicate that the development of high-risk HPV-positive cervical cancer is associated with failure of HPV-specific T-cell responses.

The humoral immune response to HPV infection is mainly directed against conformational epitopes in the variable regions of the major coat protein L1 (77). This develops slowly, and is usually weak. Indeed, seroconversion appears to occur 6–18 months after infection, and type-specific antibodies to L1 are detected in 60–70% of women who acquire HPV infection (6, 78). HPV-seroprevalence is considerably lower in men than women, and it has been suggested that HPV-seropositive women might have higher antibody levels than HPV-seropositive men (79).

IgG and IgA are the most abundant isotypes in sera from natural infections. Other HPV antigens (e.g., E1, E2, E6, L2) do not commonly induce antibody responses in patients with acute or persistent HPV infections.

Studies that have investigated whether naturally acquired HPV antibodies can protect against subsequent HPV infections have reported mixed results (80–82). More recently a systematic review and meta-analysis that included >24,000 individuals showed that natural HPV antibodies provide protection against subsequent type-specific genital HPV infections in females. However, given that the antibody titers in natural immunity are considerably lower than those observed with vaccination, and that antibody responses are preferentially induced in women and are not induced in all infected individuals, it is likely that protection through the development of natural immunity is inferior to protection obtained from HPV vaccination (83).

## HPV Vaccines

### Prophylactic Vaccines

Three prophylactic vaccines for prevention of HPV infection are available at present: a bivalent vaccine against HPV16 and HPV18 (Cervarix) that was approved in 2007; a tetravalent vaccine against HPV6, 11, 16, and 18 (Gardasil) that was approved in 2006; and a nonavalent vaccine against HPV6, 11, 18, 31, 33, 45, 52, 58 (Gardasil 9) that was approved in 2016. However, the non-avalent vaccine is the only HPV vaccine that is currently available in the USA, and it was approved for males and females from 9 to 45 years by the US Food and Drug Administration in late 2018.

Initially, the administration of the HPV vaccines was in three doses, with the more recent change to a two-dose schedule driven by evaluation of girls aged 9–13 years who had received either two or three doses. The antibody responses of the young women (aged 16–26 years) who had followed a two-dose schedule were similar to those who received all three doses (84). Therefore, in 2016, the Advisory Committee on Immunization Practice declared that there was only the need for two doses of vaccine for those under 15 years of age. However, for females who start the vaccination between 15 and 45 years old, a three-dose schedule is recommended (at 0, 1–2, and 6 months) (84, 85)^10^. Also immunocompromised patients should follow the three-dose schedule regardless of sex and age at vaccination (86).

All three of these vaccines use recombinant DNA technology and are prepared from the purified L1 protein, which self-assembles to form HPV type-specific empty shells (virus-like particles; VLPs). Only intact VLPs can generate protective antibodies, which supports the evidence that conformational epitopes of L1 are required to generate neutralizing antibodies (87).

The evidence that HPV VLP vaccines protect against high viral challenges through induction of neutralizing anti-L1 antibodies was obtained in preclinical studies in animals, which thus provided the strong rationale for development of VLP-based vaccines. In particular, in a canine model of experimentally induced oral papillomas, it was demonstrated that dogs vaccinated with the major capsid protein, L1, of canine oral papillomavirus developed antibodies against canine oral papillomavirus and became completely resistant to the viral challenge (88). Similarly, vaccination of rabbits with L1 VLPs protected them against papillomas induced by cottontail rabbit papillomavirus (89). In addition, in both of these animal models, passive transfer of immune serum protected the dogs and rabbits against the canine oral papillomavirus and cottontail rabbit papillomavirus challenges, respectively.

In humans, analysis of vaccine-induced antibody responses measured by several methods has demonstrated that almost 100% of vaccinated individuals generate a strong type-restricted serum antibody response to L1 VLP. These methods have included conventional enzyme-linked immunosorbent assays, competitive radioimmunoassays, competitive Luminex-based immunoassays, and pseudovirion-based neutralization assays.

Initial and follow-up studies that assessed the immunogenicity of the HPV 16/18 AS04-adjuvant vaccine in 15- to 25-year-old women showed that after vaccination, anti-HPV16 and anti-HPV18 total IgG antibodies peaked at month 7, reached a plateau between months 18 and 24, and remained constant for up to 76 months (90). Measurement of the neutralizing antibodies using pseudovirion-based neutralization assays confirmed high levels of functional antibodies as well. Then evaluation of long-term immunogenicity of the HPV16/18 vaccine in the serum of 15- to 55-year-old females revealed that the seropositivity for anti-HPV16 remained high in all of the age groups 10 years after the first vaccination. For anti-HPV18, there were more seropositive females in the 15- to 25-year-old group (99.2%) than for the 26- to 45-year-olds (93.7%) and 46- to 55-year-olds (83.8%) (90). In these studies, the anti-HPV16 and anti-HPV18 titers remained above natural infection levels in all of the age groups, and more interestingly, they were predicted to persist for more than 30 years after vaccination (91).

Comparisons of the immunogenicities of the HPV16/18 and HPV6/11/16/18 vaccines in healthy women aged 18–45 years revealed that 7 months after vaccination, the serum neutralizing antibody responses elicited by the bivalent vaccine were significantly higher than those for the HPV6/11/16/18 vaccine. The differences in these responses for HPV16 and HPV18 were maintained at month 24, and also up to month 60 in women aged 18–45 years.

Antibody titers induced by vaccination are higher than those produced by natural infection^11^. Responses to HPV vaccination is weakly influenced by gender, with higher seroconversion in males than females (99 vs. 93%), and by age, with higher antibody titers in women aged 9–15 years (92, 93). The FUTURE I trial demonstrated that the efficacy of the tetravalent HPV vaccine was 100% against condyloma in HPV-naïve women, and 70% (vaginal condyloma) to 78% (vulvar condyloma) in the overall population. The efficacy of the non-avalent vaccine is comparable to that of the tetravalent vaccine against condyloma (94). Prophylactic HPV vaccines show excellent protection against high-grade CIN (i.e., CIN2, CIN3) and adenocarcinoma *in situ* for HR-HPV–naïve women. In particular, the non-avalent vaccine showed the highest efficacy for prevention of onset of CIN1 (relative risk reduction, 98.9%), CIN2 (97.1%), and CIN3 (100%) neoplasia (95).

Data for vaccine prevention against AIN are more limited. In the Guanacaste study, the tetravalent HPV vaccine prevented HPV16/18 infection in anal anatomic sites in 84% of women who were HPV-seronegative at baseline (96). Palefsky reported 77.5% prevention of AIN among HPV-naïve men aged 16–26 years who had sex with men (MSM) (97). The tetravalent vaccine also protects heterosexual naïve men from both anogenital HPV infections and HPV lesions, with an efficacy against infections and associated lesions of >90% (98). Also a Finnish randomized trial reported significant reduction of genital HPV infections in men following HPV16/18 vaccine administration (99).

For oropharyngeal cancer prevention, a risk reduction of 93.3% for precursor lesions of HPV-induced oral cancer was reported for the Guanacaste study (96). However, further studies are needed to demonstrate the efficacy of these vaccines on oropharyngeal cancer development.

### Therapeutic Vaccines

The therapeutic vaccines differ from the prophylactic vaccines as they are aimed at the generation of cell-mediated immunity, rather than neutralizing antibodies. Although prophylactic vaccines can prevent HPV infections in 100% of cases, and precancerous cervical lesions (i.e., CIN) caused by the HPV genotypes included in the vaccine, HPV-related lesions remain a public problem worldwide for several reasons: (i) only 8% of low and middle income countries have introduced HPV vaccination programs^12^; (ii) HPV types that are not included in vaccines might be responsible for cancers (100); (iii) the cost of requirements for a cold chain and the absence of sanitary infrastructure limits HPV vaccine deployment in developing countries; and (iv) HPV vaccines are recommended for young women (9–26 years old), and as women older than 26 years are not vaccinated, they can develop cancers. It is also estimated that the impact of HPV vaccination on cancer incidence might not be appreciated for at least 20 years from any mass vaccination.

Currently, the treatment of high-grade disease (CIN2-3) includes electrosurgical excision of the transformation zone, with carbon dioxide lasers or knives used to perform conization, where the entire transformation zone is removed (101, 102) (Table 1). Incomplete excision, however, can occur, and HPV transformed cells can remain, which will facilitate recurrent neoplasia. Hence, there is the need for a therapeutic vaccine that can fully eliminate malignant cells.

**Table 1:** Conventional treatment of HPV-related cancers.

**Cancer HPV related lesion**	**Conventional treatment**
High-grade CIN	1. Loop electrosurgical excision procedure. 2. Cold knife. 3. Cone biopsy. 4. Electrofulgaration. 5. Cold-coagulation. 6. Cryotherapy.
Cervical cancer	1. Conization. 2. Radical hysterectomy. 3. Chemotherapy.
Vulvar intraepithelial neoplasia (VIN) and vulvar cancer	1. Surgical excision. 2. Topical agents (imiquimod). 3. Photodynamic therapy.
AIN and anal cancer	1. Ablative. 2. Chemotherapy (5-fluoracil, imiquimod, cidofovir).
PeIN and penile cancer	1. Surgical treatment. 2. Cisplatinum-based regimen.

CIN, cervical intraepithelial neoplasia; AIN, anal intraepithelial neoplasia; PeIN, penile intraepithelial neoplasia.

The aim of a therapeutic vaccine against HPV is to induce *in-vivo* virus-specific T-cell responses against established HPV infections and lesions. For therapeutic vaccination to deliver unequivocal clinical benefits, improvements must be achieved at two levels: by maximizing the induction of T-cell responses with optimal amplitude, specificity and effector profile; and by ensuring that vaccine-induced T-cells can reach the tumor site and perform their functions without restraint (103).

Among the HPV proteins, the E6 and E7 oncoproteins are considered to be almost ideal targets for immunotherapy of cervical cancer, as these proteins are essential for the onset and evolution of malignancy, and are constitutively expressed in both premalignant and invasive lesions, while being absent in healthy cells (104). E6 and E7 have therefore been included in most therapeutic vaccines developed to date. Usually, a DNA sequence that encodes a fusion protein of E6 and E7 is inserted into a vector, and mutations are introduced into the regions that are responsible for the E6 interactions with p53, and the E7 interactions with pRB, to reduce their oncogenic power. The E1 and E2 viral proteins are also attractive candidates for therapeutic vaccines that target early viral infections, as they are highly expressed before viral genome integration (105).

Several strategies have been investigated for HPV therapeutic vaccines designed to enhance CD4^+^ and CD8^+^ T-cell responses, including genetic vaccines (e.g., DNA/ RNA/ virus/ bacterial), and protein-based, peptide-based or dendritic-cell-based vaccines. Among the bacterial vectors, live attenuated *Listeria monocytogenes* has been used to generate a promising HPV therapeutic vaccine. *L. monocytogenes* is considered a potent vaccine vector because it enters professional antigen-presenting cells and induces antigen-presenting cell maturation, and strong innate and adaptive immunity (106). In addition, *L. monocytogenes* grows very efficiently *in vitro* and lacks lipopolysaccharides, which are a major toxicity factor with Gram-negative bacteria (104). The safety of a recombinant live attenuated *L. monocytogenes* secreting E7 as a fusion protein joined to non-hemolytic listeriolysin O (*Lm*-LLO-E7) was demonstrated in a phase I clinical study that was conducted with 15 patients with late-stage metastatic cervical cancer (107). Evaluation of the efficacy of *Lm*-LLO-E7 (also known as ADXS11-001) in a prospective phase II clinical trial as second-line and third line for patients with recurrent metastatic cervical cancer showed that 12-month overall survival was 38%, which exceeded the historical overall survival of such patients, of 25%. A phase III clinical trial of *Lm*-LLO-E7 for high-grade cervical cancer is being conducted at the time of writing (see NCT02853604).

Encouraging data have also been obtained in clinical studies that have tested DNA-based vaccines. DNA vaccination consists of direct introduction into tissues of a plasmid that contains the DNA sequence that encodes the antigen(s) against which an immune response is sought. This relies on *in-situ* production of the antigen(s) as a result of the transfection of antigen-presenting cells and non-antigen-presenting cells, with the presentation of the expressed antigen(s) by both MHC class I and class II molecules. Furthermore, this results in activation of all three arms of the adaptive immune response (i.e., helper T cells, cytotoxic T cells, antibodies).

However, although DNA vaccines have been shown to induce balanced CD4^+^ and CD8^+^ T cells as well as humoral immune responses in small animal models, clinical data from multiple studies have demonstrated that they induce poor T-cell responses (108).

Many strategies to facilitate antigen processing and presentation, and also antigen delivery, have been adopted to ameliorate the immunogenicity of DNA vaccines against HPV (109–111).

A phase I study was carried out using the DNA vaccine VGX-3100 that consists of a mixture of two plasmids that encode the optimized consensus of the E6 and E7 genes of HPV genotypes 16 and 18. These were delivered via intramuscular injection, followed by electroporation, with 18 patients who had been previously treated for cervical intraepithelial neoplasia (CIN2/3). This study showed that 78% of the patients developed CD8+ T-cell responses, and 100% showed antibody positivity to at least two vaccine antigens (112). Notably, the peripheral blood T-cell responses elicited by VGX-3100 were an order of magnitude greater than naturally occurring responses, and a log unit greater than those previously reported for HPV therapeutic vaccines (112).

In 2015, the efficacy, safety, and immunogenicity of VGX-3100 was assessed in a phase II clinical trial in patients with CIN2/3. In the per-protocol analysis, 30.6% of the placebo recipients and 49.5% of the VGX-3100 recipients showed histological regression. Concomitant histopathological regression and viral clearance occurred in 14.3% of placebo recipients compared with 40.2% of vaccinated recipients (113). *Post-hoc* immunological analysis here demonstrated that VGX-3100 elicited significantly increased frequency of T-cell responses against HPV16/18 E6 and E7, and that the magnitude of the T-cell response against E6 was associated with clinical outcome. Humoral immune responses were also lower in placebo recipients than in VGX-3100 recipients, and the antibody responses against HPV16, HPV18, and E7 were significantly higher in the patients who had concomitant histopathological regression and viral clearance, compared to those who did not (113). A phase III clinical trial of VGX-3100 for women with CIN was initiated in 2017, and it is expected to end in 2021 (see NCT03185013).

Viral vectors including adenoviruses, adeno-associated viruses, alphaviruses, and vaccinia viruses (e.g., modified vaccinia Ankara virus; MVA) can be used to express the E2, E6, and E7 oncoproteins, and they can stimulate CD4^+^ and CD8^+^ T-cell responses. A MVA vector was used to produce the Tipapkinogen Sovacivec vaccine, which includes three exogenous genes that encode the human cytokine interleukin-2, and non-oncogenic E6 and E7. This vaccinia virus can induce interferon-α production and express HPV16 E6 and E7, which are presented by dendritic cells to activate naïve T cells in lymph nodes. At a follow-up of 2.5 years, compared to the placebo cohort at 10% viral clearance, the administration of Tipapkinogen Sovacivec vaccine provided complete resolution for 24% of patients with CIN2/3, irrespective of their HR-HPV baseline infection (i.e., HPV16, 18, 31, 33, 35, 39, 45, 52, 56, 58, 59, or 68). However, despite this significantly improved HPV viral clearance with this vaccine, it has still not been licensed for clinical use because of the modest efficacy (104).

Finally, a vaccine designed on recombinant MVA that contained the bovine papillomavirus E2 protein (MVA E2) was used to treat HPV-induced ano-genital intraepithelial lesions. A phase III study showed that 90% of female patients had complete elimination of lesions after treatment with MVA E2, with 100% seen for men. All of these patients treated with MVA E2 developed antibodies against the MVA E2 vaccine and generated a specific cytotoxic response against the papilloma-transformed cells (114).

Interestingly, novel vaccination strategies aimed to maximize systemic as well as genital resident memory T cell responses to treat sexually transmitted infections and human papilloma virus neoplasia are being developed. In this context several studies have investigated the effect of either the topical delivery of host and pathogen derived immunomodulatory molecules or the delivery route of immunization in the induction of cervicovaginal long lived CD8+ T cell responses (115).

## HPV Prophylactic Vaccines Used as Therapeutic Vaccines

The treatment of premalignant lesions (CIN2,3) by LLEP or conization sometimes fails to prevent lesion recurrence (116–118). This is often linked to incomplete excision of transformation zone consciously carried out by gynecologists. In fact, evidence shows that large excision of the cervix can compromise cervix integrity and can cause adverse neonatal outcome with preterm risk (119, 120). Moreover, recurrence risk is greater in presence of HR HPV infection (116, 121).

A systematic review of studies of the treatment of high-grade lesions (HSIL/ CIN2-3) reported that a median of 28% of the women remained positive for oncogenic HPV types 3 months after treatment. A decrease in this HPV persistence was seen during follow-up, as it fell to 21% after 6 months (102). Also, higher risks for the development of cervical and vaginal neoplasia have been reported for women who had previously been treated for CIN3, in comparison to the general female population, with this higher risk persisting for 20–25 years, and possibly longer (122). The risk of cervical cancer after treatment also increases with age. A large study with long-term follow-up for women treated for CIN3 reported standard incidence and mortality ratios (i.e., treated vs. placebo) for cervical and vaginal cancers of 10.58 and 7.60, respectively, for women aged 60–69 years, and 2.03 and 1.52, respectively, for women aged 30–39 years (123). Also, women who had previously reported CIN3 lesions showed greater probability of developing other HPV-related neoplasia of the genital tract (e.g., vaginal, vulvar, anal) or the oropharyngeal district (124).

As no vaccine has yet been licensed for therapeutic use, the prophylactic vaccines have been tested in several trials to determine their effectiveness for prevention of HPV disease recurrence or reinfection after CIN2-3 treatment. The recurrence for MSM who undergo treatment for high-grade anal intraepithelial neoplasia (HGAIN) is particularly high, as 50% show recurrence within 1 year (123). This makes it essential to find a treatment that can reduce the development of high-grade lesions in treated patients. In 2011, the effectiveness of the tetravalent HPV vaccine for the prevention of recurrent HGAIN was evaluated in HIV-negative, self-identified MSM with a history of biopsy-proven and treated HGAIN. In the 340.4 person-years of follow-up, 30.7% of the non-vaccinated patients developed recurrent HGAIN, compared to 13.6% of the vaccinated patients. Among these patients who were infected with HR-HPV types, the tetravalent vaccine was associated with significantly decreased risk of recurrent HGAIN at 2 years from study entry (hazard ratio, 0.47). To explain the partial effectiveness of the tetravalent vaccine in this study, it was speculated that some of these patients might have developed diseases that were related to the HPV genotypes not covered by the tetravalent vaccine or to multiple HPV infections. Further, some HGAIN might not have been identified and treated before the vaccinations, or the viral integration into the host genome had already occurred. Unfortunately, these aspects were not investigated.

In 2013, Kang et al. investigated the effectiveness of the tetravalent HPV vaccine to prevent recurrence of CIN2-3 in patients with high-grade CIN treated by the loop electrosurgical excision procedure (125). Recurrence was seen for 7.2% of the non-vaccinated patients and by 2.5% of the vaccinated patients. In patients infected with HPV16 and/or HPV18, 8.5% of the non-vaccinated patients and 2.5% of the vaccinated patients developed recurrent disease related to these HPV types. Although encouraging, these data indicate that the prophylactic HPV vaccine had weak activity against such HPV16/18-related high-grade lesions. Recently, a prospective clinical project, the SPERANZA study, was carried out to determine the effectiveness of the tetravalent vaccine for reduction of the risk of clinical relapse in women treated for CIN2 (126). Overall, 344 women were included in the study, and 6.4% of the non-vaccinated women showed clinical disease recurrence, while for the vaccinated women, there was only 1.2% recurrence. Vaccination here was associated with significantly reduced risk of subsequent HPV-related high-grade CIN after cervical surgery, at 81.2%. For the non-vaccinated women, the recurrent clinical disease was attributed to HPV11, 16, 18, 31, 33, 45, 53, 82, while for the vaccinated women, the two cases of clinical disease recurrence were associated with HPV33 and HPV82. In this study, about 40% of the patients enrolled were >36 years old, although neither the age range nor the age of women with recurrent clinical disease were reported, and thus it cannot be determined if the efficacy of the tetravalent HPV vaccine was influenced by the age of the patients at the time of their vaccination. At variance with this, a study by Hildesheim et al. included 1,711 women with carcinogenic human HPV infection and 311 women who received loop electrosurgical excision for cervical precancer. Here, there was no evidence that HPV16/18 vaccination alters the fate of an HPV infection present at the time of vaccination, or the rates of cervical infections and lesions after loop electrosurgical excision. For these HPV16/18 infections, in the cohort of women with HPV infection but without precancer, the efficacy of clearance was 5.4%, with progression to CIN1 seen for 15.5%, and to CIN2, for 0.3%. Moreover, after the loop electrosurgical excision, the vaccination had no significant effects on HPV16/18 infections and/or HPV16/18-associated cytological and histological lesions (127).

The data obtained on the efficacy of the tetravalent HPV vaccine for the prevention of anal condylomas are, however, more encouraging (128). Three hundred and thirteen MSM (mean age, 42 years) were enrolled for a median of 981 days. During the follow-up, condyloma developed in 18.8% of non-vaccinated patients, and in 8.6% of vaccinated patients. Moreover, several clinical studies have demonstrated activity for HPV vaccination in the treatment of genital warts (129–131).

Altogether, these data suggest that there is possibility that prophylactic vaccines reduce the risk of HSIL recurrence in previously infected patients, although the exact protective mechanisms in infected individuals is not understood. The high risk of recurring infections is consistent with either auto-inoculation across anatomic sites or new inoculation or episodic reactivation of latent infection. As HPV vaccines prevent infection by induction of L1-specific antibodies that block viral entry, and L1 is not generally expressed during the oncogenic process, it is expected that these vaccines will be effective in the prevention of auto-inoculation or new infections.

The greater effectiveness obtained with prophylactic vaccines in the prevention and regression of genital warts might be related to the integration state of the HPV genome. In genital warts, the virus is not generally integrated into the host genome, and therefore viral particles are produced. In this case, the prophylactic vaccines that block viral entry through induction of L1-specific antibodies can prevent reinfections, which will favor the elimination of the virus. Conversely, in high-grade lesions, the virus genome is often integrated into the host genome, and infected cells do not express L1 and do not produce viral particles. Thus, as transformed cells are frequently in the basal layer of the derma, they will not be recognized by vaccine-induced antibodies, which are ineffective in the control of the disease course.

Furthermore, there are some cases in the treatment of HPV-related cancers where the use of prophylactic vaccines might not be recommended:

(1) Anal and cervical cancers that are not attributable to the HPV types that are included in the non-avalent HPV vaccine. Several studies have demonstrated that half of the HPV infections in MSM are caused by HPV types that are not included in the non-avalent HPV vaccine (132, 133) (Table 2). Here, over 2 years of observation, only about 30% of HIV-positive MSM had incidents of HR-HPV infections that were covered by the non-avalent vaccine (134). This situation can be also observed for women (Table 2).

**Table 2:** Detail of cervical and anal samples from HIV positive patients with squamous intraepithelial lesions and HPV DNA negative or positive for HPV types that are not included in nonavalent vaccine.

		**N. of cases (%) Pt HPV- 9v Neg**	**N. of cases (%) Pt HPV DNA Neg**
Cervical samples	LSIL (*n* = 231)	72 (31.2)	70 (30.3)
	HSIL (*n* = 55)	17 (30.9)	6 (10.9)
Anal samples	AIN 1 (*n* = 18)	9 (50)	3 (16.7)
	AIN 2 (*n* = 7)	5 (71.4)	0
	AIN 3 (*n* = 1)	1	0

*Pt, patient; LSIL, Low Grade Squamous Intraepithelial Lesion; HSIL, High Grade Squamous Intraepithelial Lesion; AIN, Anal intraepithelial neoplasia; 9v, nonavalent vaccine. These data are partially presented in CME event “Novità nel campo dell'infezione da HPV,” Rome, 20th June 2018 INMI L Spallanzani IRCCS*.

(2) HPV DNA-negative cervical tumors. Over recent decades, several studies have reported that some cervical cancers are HPV-negative (135–139). Often, HPV DNA negativity is due to the sensitivity of the methods used in the HPV DNA detection, and so samples that have tested as HPV-negative might show as HPV-positive when retested with more sensitive assays (e.g., nested PCR) (136). The Cancer Genome and Molecular Characterization of Cervical Cancer Study used next-generation sequencing to characterize primary cervical cancers, and it established that 5% of the specimens were HPV-negative. This subset of HPV DNA-negative cancers is mainly observed among adenocarcinoma cancers, and predominantly in gastric-type adenocarcinomas. The pattern of immunostaining of gastric-type adenocarcinomas shows strong and diffuse positivity for MUC-6 and HIK1083 antibodies, which recognize epitopes of gastric pyloric glycoproteins, although they are p16 negative, which is a cell-cycle regulatory protein (140). Gastric-type adenocarcinomas have significantly higher rates of recurrence and mortality than HPV-positive cancers (141, 142). Furthermore, progression and regression of gastric-type adenocarcinomas are independent of HPV infection, and thus HPV vaccine administration here would be inappropriate.

## Conclusions

The data reported in this review highlight the significant efforts that have been carried out to set-up therapeutic vaccines against HPV-related malignancies. Although several approaches to produce an effective vaccine have been attempted, including the use of proteins, synthetic peptides, and viral proteins expressed in different vectors, and although some of the data appear encouraging, no therapeutic vaccines have been licensed in clinical practice yet. Recently, prophylactic vaccines have been used for treatment of recurrent forms or reinfections in subjects who have previously undergone surgical resection. However, the trials here have offered conflicting results, and vaccination did not guarantee 100% effectiveness. This is probably due to a residual burden of transformed cells that can persist after the surgical treatment, and that are not targeted by the humoral L1-specific immune response induced by the prophylactic vaccines. Although it cannot be excluded that the therapeutic potential of prophylactic vaccines could be improved by using different adjuvants or route of immunization, an additional limit in using prophylactic vaccines for therapeutic purposes is seen by the evidence that the non-avalent vaccine does not include all of the HR-HPV types. As the real extent of protection given by the non-avalent vaccine against other HPV types is not known, its use in the treatment of tumors related to these other HR-HPV types is questionable. Furthermore, for endometrial adenocarcinomas, such as gastric-type adenocarcinomas, which are HPV DNA-negative, careful virological and histological diagnosis must be made before administration of HPV prophylactic vaccines to treat HPV recurrence or reinfection.

## Author Contributions

AG and PD wrote the original version and edited versions. MC edited versions. DL and CS prepared figures and tables.

### Conflict of Interest

The authors declare that the research was conducted in the absence of any commercial or financial relationships that could be construed as a potential conflict of interest.

## Footnotes

^1^https://gco.iarc.fr/

^2^https://publications.iarc.fr/108

^3^https://monographs.iarc.fr/wp-content/uploads/2018/06/mono100B-11.pdf

^4^http://globocan.iarc.fr/old/FactSheets/cancers/cervix-new.asp

^5^https://seer.cancer.gov/archive/csr/1975_2004/

^6^https://monographs.iarc.fr/wp-content/uploads/2018/06/mono90.pdf

^7^https://monographs.iarc.fr/wp-content/uploads/2018/06/mono100B.pdf

^8^https://www.ncbi.nlm.nih.gov/books/NBK12354/

^9^http://www.seer.cancer.gov

^10^https://www.cdc.gov/hpv/dowloads/9vhpv-guidance.pdf

^11^http://www.rho.org/files/WHO_HPV_tech_info_nocover_2007.pdf

^12^https://apps.who.int/iris/bitstream/handle/10665/251810/WER9148.pdf;jsessionid=12D591BAA8A2E02CEB5223020DFC3526?sequence=1
